# BMP-9 Induced Endothelial Cell Tubule Formation and Inhibition of Migration Involves Smad1 Driven Endothelin-1 Production

**DOI:** 10.1371/journal.pone.0030075

**Published:** 2012-01-27

**Authors:** John E. S. Park, Dongmin Shao, Paul D. Upton, Patricia deSouza, Ian M. Adcock, Rachel J. Davies, Nicholas W. Morrell, Mark J. D. Griffiths, Stephen J. Wort

**Affiliations:** 1 Unit of Critical Care, Royal Brompton Hospital, National Heart and Lung Institute, Faculty of Medicine, Imperial College London, London, United Kingdom; 2 Department of Medicine, School of Clinical Medicine, Addenbrooke's Hospital, University of Cambridge, Cambridge, United Kingdom; 3 Airways Disease Section, National Heart and Lung Institute, Faculty of Medicine, Imperial College London, London, United Kingdom; University of Illinois at Chicago, United States of America

## Abstract

**Background:**

Bone morphogenetic proteins (BMPs) and their receptors, such as bone morphogenetic protein receptor (BMPR) II, have been implicated in a wide variety of disorders including pulmonary arterial hypertension (PAH). Similarly, endothelin-1 (ET-1), a mitogen and vasoconstrictor, is upregulated in PAH and endothelin receptor antagonists are used in its treatment. We sought to determine whether there is crosstalk between BMP signalling and the ET-1 axis in human pulmonary artery endothelial cells (HPAECs), possible mechanisms involved in such crosstalk and functional consequences thereof.

**Methodology/Principal Finding:**

Using western blot, real time RT-PCR, ELISA and small RNA interference methods we provide evidence that in HPAECs BMP-9, but not BMP-2, -4 and -6 significantly stimulated ET-1 release under physiological concentrations. This release is mediated by both Smad1 and p38 MAPK and is independent of the canonical Smad4 pathway. Moreover, knocking down the ALK1 receptor or BMPR II attenuates BMP-9 stimulated ET-1 release, whilst causing a significant increase in prepro ET-1 mRNA transcription and mature peptide release. Finally, BMP-9 induced ET-1 release is involved in both inhibition of endothelial cell migration and promotion of tubule formation.

**Conclusions/Significance:**

Although our data does not support an important role for BMP-9 as a source of increased endothelial ET-1 production seen in human PAH, BMP-9 stimulated ET-1 production is likely to be important in angiogenesis and vascular stability. However, increased ET-1 production by endothelial cells as a consequence of BMPR II dysfunction may be clinically relevant in the pathogenesis of PAH.

## Introduction

Bone morphogenetic proteins (BMPs) are the largest subgroup of signalling molecules in the transforming growth factor (TGF)-β superfamily. Although originally described as osteogenic factors, BMPs play crucial roles during embryonic development and determine many different aspects of cell fate such as apoptosis, proliferation, differentiation, migration, as well as angiogenesis [1]. BMPs bind to a complex of serine/threonine type I and type II bone morphogenetic protein receptors (BMPRs) on cell membranes. The type I receptor (ALK1-3 or ALK6) is activated by the type II receptor with consequent phosphorylation of downstream Smads (mothers against decapentaplegic homolog proteins). Ligands for BMPRs classically signal through receptor-mediated Smads (R-Smads) 1, 5 and 8, whereas TGF-β typically signals through Smad2 and 3, via the ALK5 receptor. Both then utilise a common partner (co)-Smad, Smad4, to form a complex that translocates to the nucleus to alter gene expression [2]. In addition to Smad signalling, BMPs may act via their receptors through “Smad independent” signalling pathways, including p38 MAPK (mitogen activated protein kinase), ERK1/2 (extracellular signal related kinase) and JNK (c-Jun N-terminal kinase) [3]. The activation of such additional pathways appears to be cell context specific. Furthermore, there is evidence for crosstalk between these pathways [4], [5].

Abnormalities in BMPR signalling are seen in various clinical conditions including pulmonary arterial hypertension (PAH). PAH is a devastating condition associated with significant morbidity and mortality [6]. Remodelling of small resistance vessels leads to a progressive increase in pulmonary vascular resistance followed by right ventricular failure [7]. Specifically, the genetic defect underlying the majority (>70%) of cases of heritable PAH is heterozygous germ-line mutations in BMPR II [8]. Similar mutations have been found in up to 26% of sporadic cases of idiopathic PAH [9], [10]. Furthermore, a mutation in BMPR II that leads to a loss of function and a reduction in BMPR II expression has now been observed in other, more common, forms of human PAH [11] and in animal models of PAH [12], [13]. Indeed, selective deletion of BMPR II or overexpression of mutant BMPR II in animal models results in the development of PAH, although usually at levels milder than in human disease [14]. Expression of the BMPR type I receptor, ALK3 is also reduced in patients with a wide variety of conditions causing PAH [15]. These findings suggest that TGF-β superfamily dysfunction is common to most, if not all, forms of PAH. Despite this, it remains unclear exactly what role BMPs themselves play in the pathogenesis of PAH.

In PAH there is also an up-regulation of the endothelin-1 (ET-1) system. ET-1 is a 21 amino acid peptide which is a vasoconstrictor and mitogen [6]. In addition, ET-1 has been shown to regulate endothelial cell migration and angiogenesis [16]. Plasma levels of ET-1 correlate positively with haemodynamic severity in patients with PAH and negatively with outcome [17], [18]. Furthermore, ET receptor antagonists (ERAs) reverse pulmonary hypertension and vascular remodeling in animal models of PAH [19]. Importantly, ERAs are licensed for the treatment of severe PAH in humans, with beneficial effects on morbidity and mortality [20]. However, the mechanisms by which ET-1 levels are elevated in PAH remain unclear.

We and others have recently shown that BMP-9 stimulates ET-1 release in vascular endothelial cells, suggesting important crosstalk between the two systems [21], [22]. However, the signalling pathways involved remain undefined as do the functional consequences in the vasculature. Indeed, it is uncertain as to whether such an interaction is relevant to the pathogenesis of PAH. In this study we sought to answer these questions more fully.

## Results

### BMP-9 increases ET-1 peptide release and gene expression in human pulmonary artery endothelial cells

**Figure 1:**
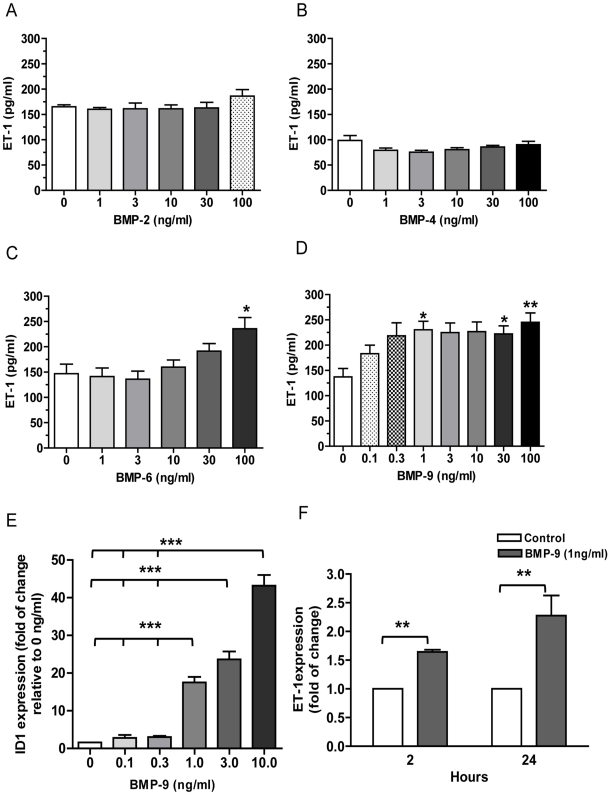
ET-1 release in response to BMPs and ET-1 mRNA expression by BMP-9 stimulation. HPAECs were grown to confluence on 96-well plates or 6-well plates and starved for 16 hrs. Cells were then stimulated with various concentrations of BMP-2, n = 4 (A), BMP-4, n = 7 (B), BMP-6, n = 5 (C) or BMP-9, n = 6 (D). Supernatants were collected at 24 hrs of treatment and ET-1 level assayed by ELISA. RNA was extracted from cells treated with increasing concentration of BMP-9 for 24 hrs. Id-1 gene expression was determined by qRT-PCR and normalised to the average of 2 housekeeping genes and shown relative to expression of non-stimulated controls, n = 3 (E). RNA was extracted from cells treated with 1 ng/ml BMP-9 at 2 hrs and 24 hrs time points and ET-1 gene expression determined by qRT-PCR. ET-1 was normalised to the average of 2 housekeeping genes and shown relative to expression of non-stimulated controls at 2 hr and 24 hs, n = 4 (F). Data are presented as mean ± SEM. *p<0.05, **p<0.01, *** p<0.001.

Of the BMPs studied (BMP-2, -4, -6 and -9), only the addition of BMP-6 and -9 resulted in a significant increase in ET-1 released by human pulmonary artery endothelial cells (HPAECs) over 24 hrs (Figure 1A–D). However, the concentrations of BMP-9 which stimulated ET-1 release were considered more “physiological” than those of BMP-6, and thus further experiments only involved BMP-9. From the concentration response shown in Figure 1D we chose a concentration of 1 ng/ml for further experiments [23]. As a positive control we examined Id-1 expression over a similar concentration range of BMP-9 (Figure 1E). The results are in keeping with previous publications [2]. In addition, BMP-9 had a similar effect on ET-1 release from human lung microvascular endothelial cells (Text S1 and Figure S1), suggesting the response is not specific to HPAECs. Importantly, BMP-9 treatment did not alter cell number over the 24 hrs period, as measured by MTT and CyQUANT assay (data not shown). Furthermore, BMP-9 stimulated a 2 fold and 3.6 fold increase in prepro ET-1 transcription at 2 hrs and 24 hrs, respectively, as determined by real-time quantitative RT-PCR (Figure 1F).

### BMP-9 stimulated transcription and release of ET-1 in HPAECs is Smad1 dependent, but independent of Smad4

**Figure 2:**
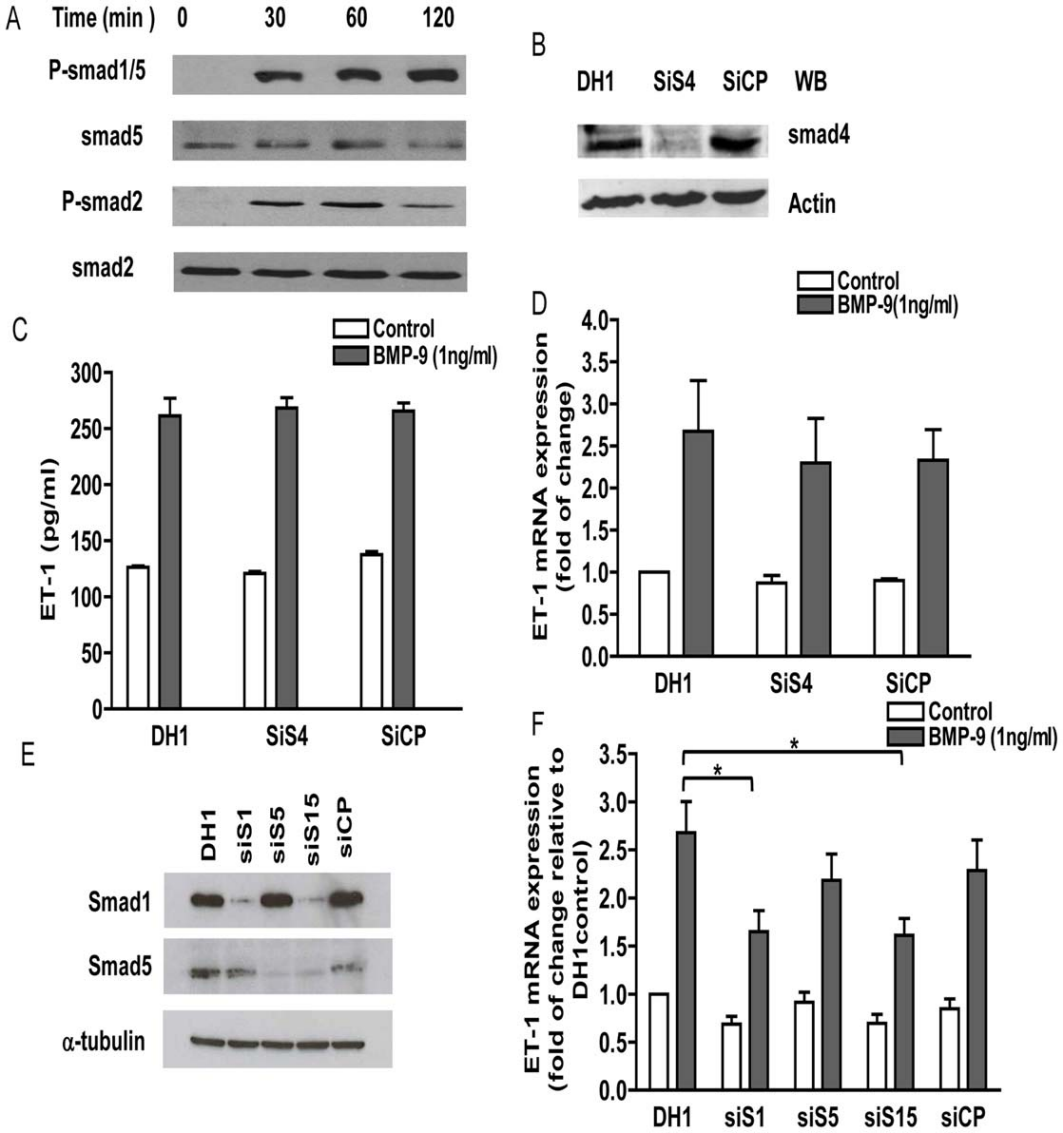
Effects of Smad signalling pathway on BMP-9 induced prepro ET-1 mRNA transcription and ET-1 release. HPAECs were grown to confluence in 6-cm dishes, starved for 16 hrs and then stimulated with BMP-9 (1 ng/ml). Phosphorylation of Smad1/5 and Smad2 were determined by western blot (A). After transfected with Smad4 specific or non-targeting siRNA and starved for 16 hs, HPAECs were stimulated with BMP-9 (1 ng/ml). The knockdown efficiency was determined by western blotting (B). After siRNA treatment and BMP-9 stimulation, supernatants and cells were collected at 24 hrs (6-well plates) for determination of ET-1 peptides by ELISA, n = 4 (C) and mRNA levels by qRT-PCR, n = 4 (D). HPAECs were transfected with Smad1, Smad5 or non-targeting siRNA. Specific Smad1 and Smad5 knockdown were confirmed by qRT-PCR (not shown) and western blotting (E). After siRNA treatment, HPAECs were starved and stimulated with BMP-9 (1 ng/ml) for 24 hrs. RNA was extracted and gene expression determined by qRT-PCR. ET-1 was normalised to β-actin and is expressed relative to cells exposed to DH1 only, n = 5 (F). Data are presented as mean ± SEM. **p<0.01.

To define the signaling pathways leading to ET-1 release by BMP-9 stimulation, we first investigated Smad protein phosphorylation by western blot. As shown in Figure 2A, Smad1/5 and Smad2 were phosphorylated after 30 min of BMP-9 stimulation. To determine the importance of Smad activation, including co-Smad4, we employed small interfering RNA (siRNA) knock-down. SiRNA silencing reduced Smad1, 4 and 5 protein levels by more than 80% compared to DharmaFECT 1 transfection agent alone (DH1) and negative control siRNA (CP) (Figure 2B). In comparison to cells treated with BMP-9 and the controls, a reduction in BMP-9 mediated prepro ET-1 mRNA transcription was observed in cells transfected with Smad1 siRNA alone (61.7±9.8% reduction) or in combination with Smad5 siRNA (67.5±8.8%). In contrast, Smad5 siRNA alone reduced the ET-1 response by only 33.7±9.4% (Figure 2E&F). To confirm specificity of Smad1 silencing we performed a rescue experiment with a lentiviral mouse Smad1 construct. The inhibition of BMP-9 stimulated ET-1 release was reversed by the Smad1 construct (Text S1 and Figure S2A). In addition siRNA silencing of Smad2 had no effect on prepro ET-1 mRNA transcription (data not shown). Unexpectedly, co-Smad4 siRNA silencing had no effect on BMP-9 induced prepro ET-1 transcription or mature peptide release (Figure 2C&D). We did not observe any changes in cell morphology after transfection of siRNAs over the time period of the experiments (Text S1 and Figure S3). Overall these results indicate that BMP-9 induced prepro ET-1 mRNA transcription (and mature peptide release) is Smad1 dependent, but independent the classical Smad signalling cascade via co-Smad4.

### p38 MAPK, ERK and JNK phosphorylation in HPAECs: effect of BMP-9

**Figure 3:**
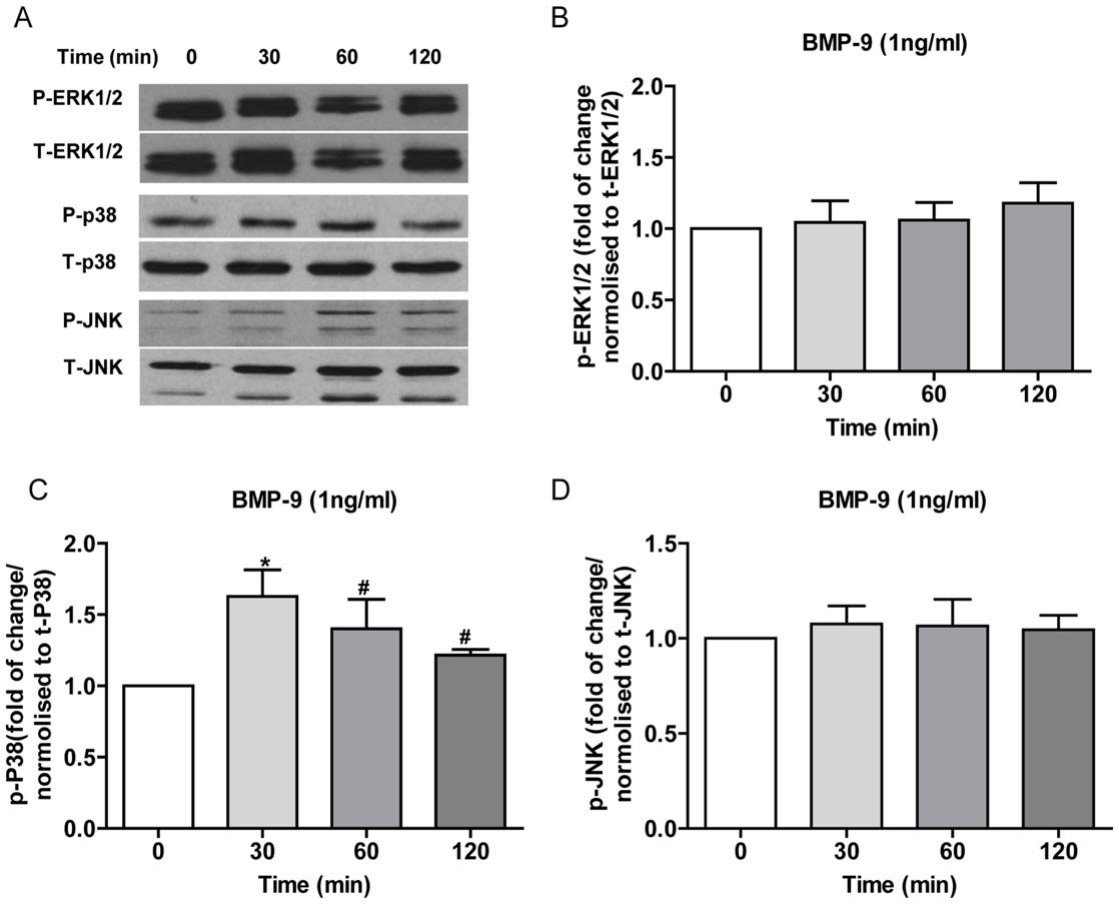
ERK1/2, JNK and p38 MAPK phosphorylation in HPAECs stimulated with BMP-9. HPAECs were grown to confluence in 6-cm dishes, starved for 16 hrs and then stimulated with BMP-9 (1 ng/ml). Phosphorylation of ERK1/2, JNK and p38 MAPK were determined by western blot (A), and quantified by densitometry (B, C and D). Data are presented as mean ± SEM. n = 3, #p>0.05; *p<0.05, one -way ANOVA, control vs. BMP-9 stimulation.

To investigate whether Smad independent signalling pathways may be involved in BMP-9 induced ET-1 release from HPAECs we looked at phosphorylation of ERK1/2, JNK and p38 MAPK at baseline and following BMP-9 stimulation. Consistent with our previous findings [24], ERK1/2 was constitutively phosphorylated at baseline in HPAECs, and showed no detectable change following BMP-9 stimulation (Figure 3A&B). There was minimal phosphorylation of JNK at baseline with no significant increase after BMP-9 stimulation (Figure 3A&D). Similar to ERK1/2, p38 MAPK was phosphorylated constitutively at baseline. In contrast, compared to non-stimulated cells, p38 MAPK phosphorylation was modestly increased 1.6 fold, 1.4 fold and 1.2 fold at 30 min, 60 min and 120 min respectively, following addition of BMP-9 (Figure 3A&C).

### Regulation of ET-1 by BMP-9 in HPAECs is p38 MAPK dependent

**Figure 4:**
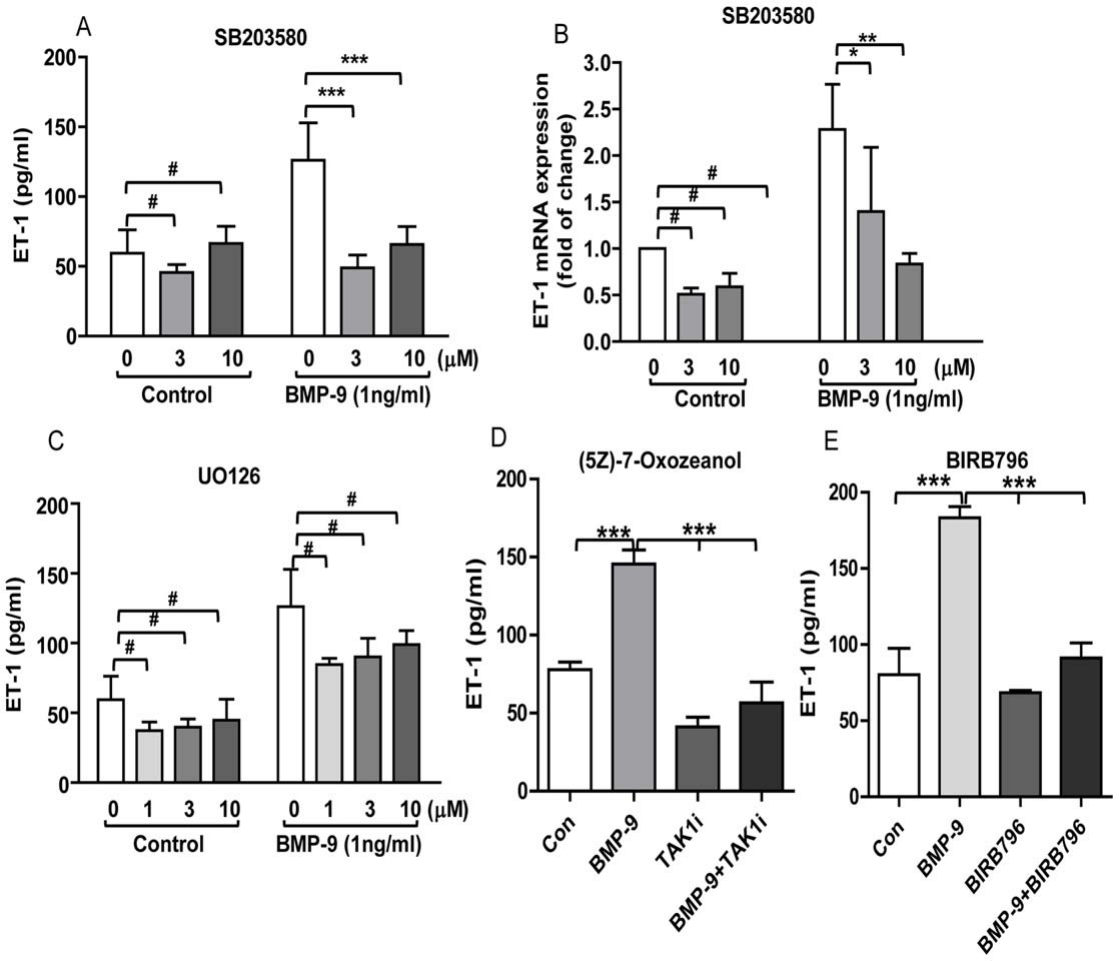
Effects of p38 MAPK and ERK1/2 inhibition on BMP-9 stimulated prepro ET-1 mRNA transcription and ET-1 release. HPAECs were grown to confluence, starved for 16 hrs and then pre-incubated with 1–10 µM SB203580, 1–10 µM UO126, 0.5 µM (5Z)-7-Oxozeanol or 1 µM BIRB796 for 60 min before adding BMP-9 (1 ng/ml) for another 24 hrs. Supernatants and cells were collected for determination of ET-1 by ELISA n = 4 (A, C, D, E), and prepro ET-1 mRNA levels by qRT-PCR, n = 3 (B). Data are presented as mean ± SEM. #p>0.05; * p<0.05; **p<0.01; ***p<0.001.

To determine whether ET-1 regulation by BMP-9 was dependent on p38 MAPK, we used the specific pharmacological inhibitor SB203580. SB203580 (10 µM) completely inhibited BMP-9 induced prepro ET-1 mRNA transcription and ET-1 peptide release (Figure 4A&B). There was no change in cell viability as determined by MTT assay (results not shown). To further confirm these finding we used an alternative, highly specific, p38 MAPK inhibitor BIRB796 (1 µM) and the upstream TAK-1 inhibitor (5Z)-7-Oxozeanol (0.5 µM) at concentrations considered to be specific to inhibition of the MAPK pathway [25], [26]. Both inhibitors abolished BMP-9 stimulated ET-1 release from HPAECs over a 24 hrs period (Figure 4D&E). The ERK1/2 inhibitor UO126 (1–10 µM) caused a small non-significant reduction in ET-1 peptide release after BMP-9 stimulation, but a similar reduction was seen with baseline ET-1 levels (Figure 4C). Finally, we found that the JNK inhibitor (SP600125) was toxic to HPAECs as determined by MTT (data not shown) and therefore the results are not included.

### BMP-9 induced ET-1 release is only partially mediated by ALK1 and BMPR II receptors. Basal prepro ET-1 mRNA transcription and ET-1 release is increased by BMPR II knock-down

**Figure 5:**
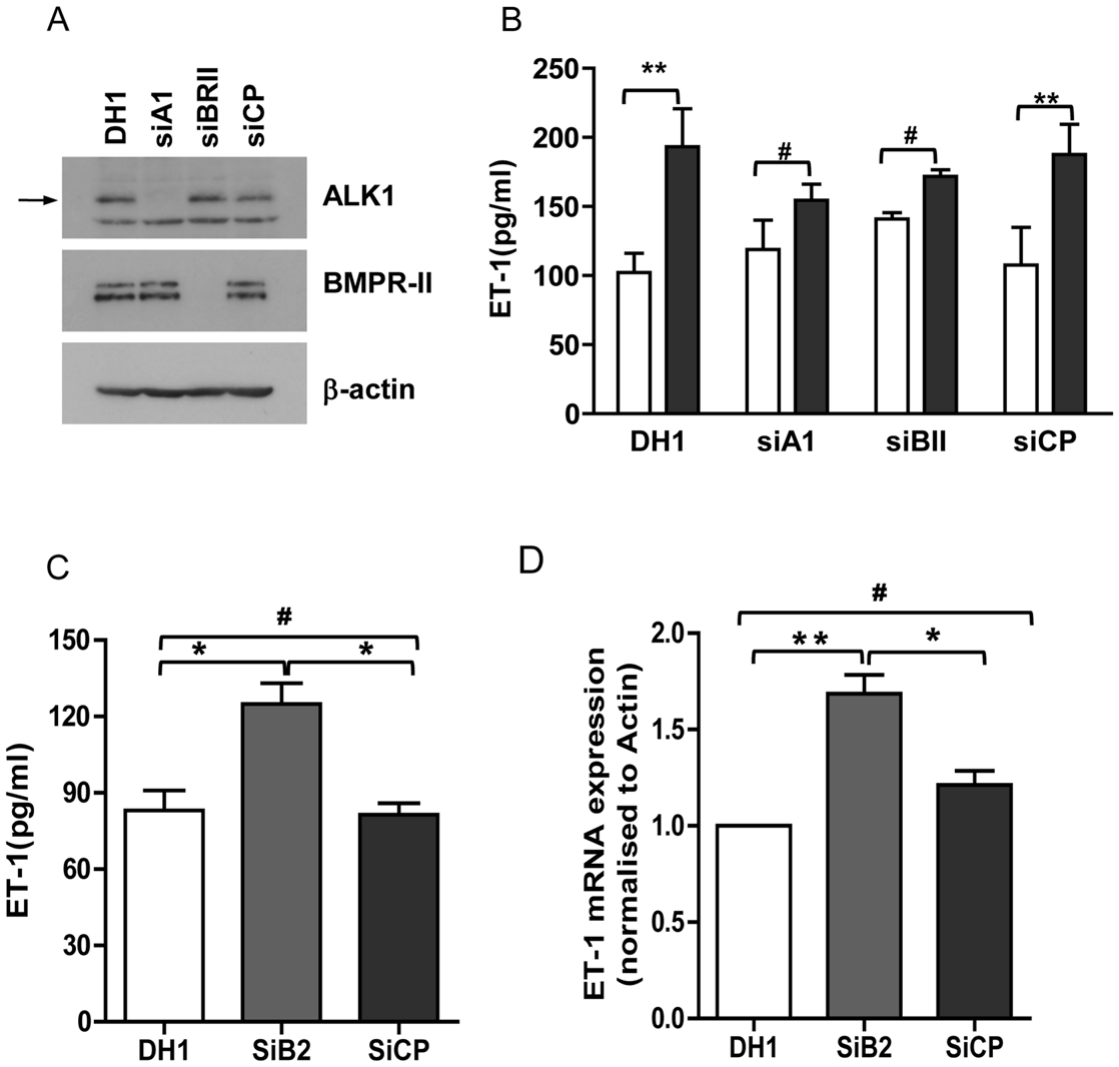
Effects of ALK1 and BMPR II siRNA on BMP-9 induced ET-1 release. HPAECs were transfected with ALK1, BMPR II specific or non-targeting siRNA. Cells were starved for 16 hrs and stimulated with BMP-9 (1 ng/ml) for 24 hrs. Knock-down efficiency was determined by western blotting. The arrows denote ALK1 (62 kDa) and BMPR II (doublet 190 kDa and 145 kDa) (A). Supernatants were collected at 24 hrs post-stimulation for determination of ET-1 release by ELISA (B&C). RNA was extracted from HPAECs treated with BMPR II specific siRNA and gene expression was determined by qRT-PCR (D). Data are presented as Mean ± SEM, n = 4. #p>0.05; **p<0.01.

ALK1 and BMPR II are the main receptors that bind BMP-9 in endothelial cells [2]. To establish the role of these receptors in BMP-9 stimulated ET-1 release in HPAECs we performed siRNA knock-down of the receptors. SiRNA knock-down efficiently reduced ALK1 and BMPR II protein expression by greater than 80% (Figure 5A). Knock-down of ALK1 or BMPR II resulted in a moderate but incomplete reduction in BMP-9 stimulated ET-1 release (Figure 5B). To confirm specificity of BMPR II silencing we performed a rescue experiment with a lentiviral mouse BMPR II construct. The inhibition of BMP-9 stimulated ET-1 release was reversed by the BMPR II construct (Text S1 and Figure S2B). In these experiments it appeared that there was an increase in basal release of ET-1 with BMPR II knock-down. Therefore we repeated these experiments and also looked at the effect of BMPR II knock-down on prepro ET-1 mRNA transcription. Consistent with the earlier results we found a significant increase in prepro ET-1 mRNA transcription (47.4±8.94% increase over control siRNA (CP)) and also a significant increase in mature peptide release (55.16±15.34% increase over control siRNA(CP)) (Figure 5C&D).

### Role of endogenously released ET-1 in BMP-9 induced tubule formation

**Figure 6:**
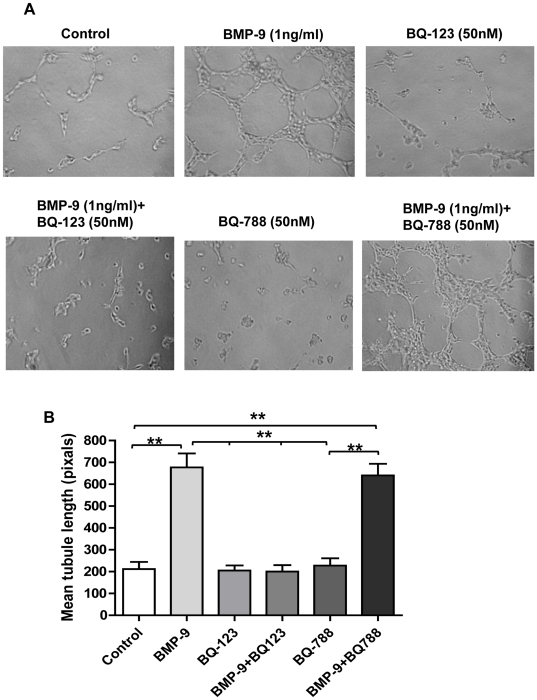
Effect of BMP-9 induced ET-1 release on HPAEC tubule formation. HPAECs were seeded onto matrigel and tubule formation capacity analysed as described in the materials and methods section. Representitive images of 3 independent experiments of tubule formation are shown in (A). The average length of the tublule networks in the various conditions shown was quantified using Image J (NIH) and represented graphically in (B). Data are presented as mean ± SEM, n = 3. **p<0.01.

**Figure 7:**
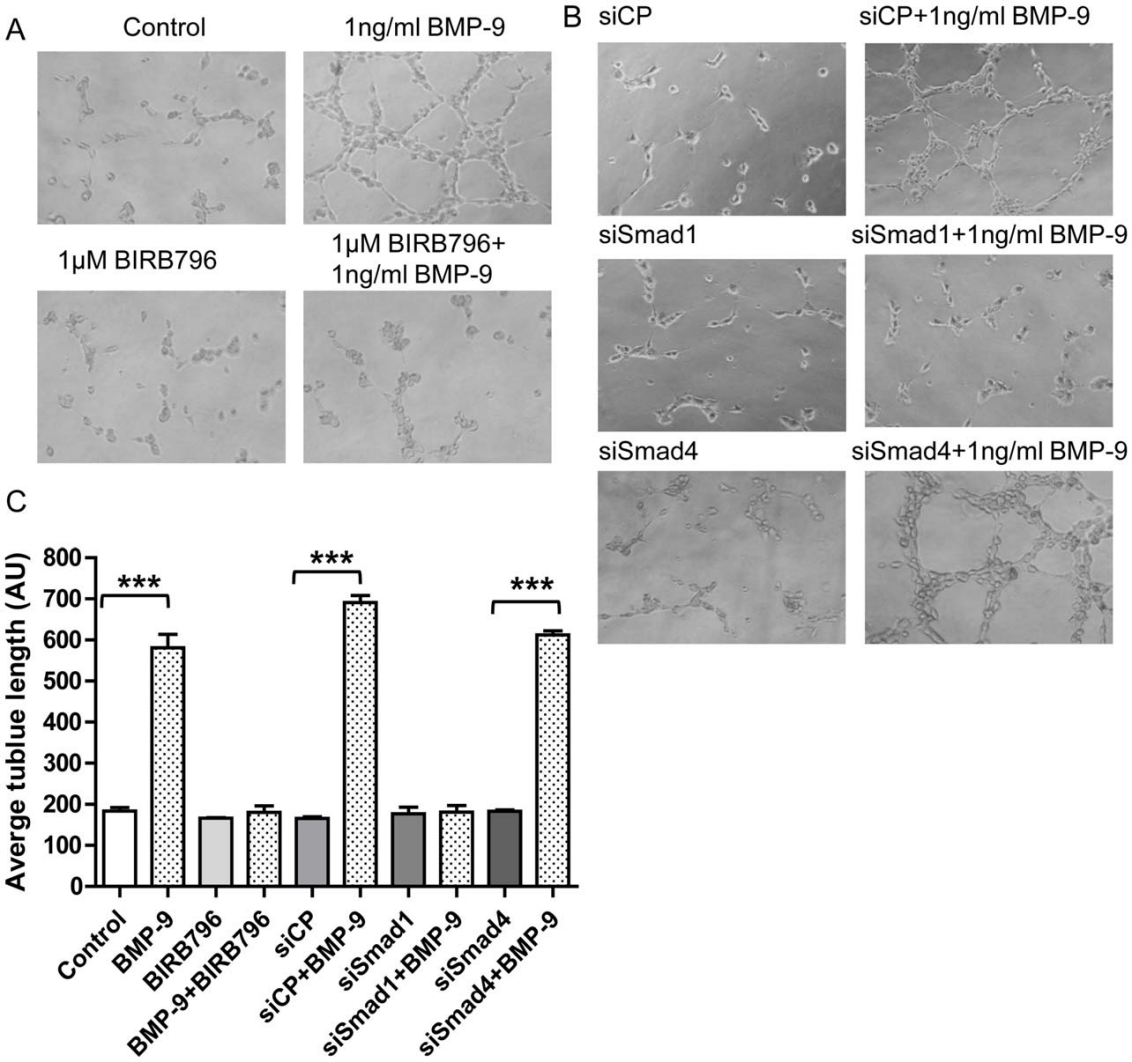
Effect of p38 MAPK inhibition and Smad1/Smad4 knock-down on BMP-9 induced HPAEC tubule formation. HPAECs or HPAECs transfected with negative control (CP), Smad1 and Smad4 siRNA were seeded onto matrigel and tubule formation capacity analysed as described in the materials and methods section. Representitive images of 3 independent experiments of tubule formation are shown in (A&B). The average length of the tublule networks in the various conditions shown was quantified using Image J (NIH) and represented graphically in (C). Data are presented as mean ± SEM, n = 3. ***p<0.001.

To investigate whether BMP-9 stimulated ET-1 release had a functional role in HPAECs we looked at BMP-9 stimulated tubule formation using matrigel. After 4 hrs of BMP-9 stimulation, HPAECs formed a complex tubule network as shown in Figure 6A. BMP-9 stimulated tubule formation was inhibited by the ET_A_ receptor antagonist BQ-123 (50 nM), but not by the ET_B_ receptor antagonist BQ-788 (50 nM) (Figure 6A&B). Both compounds alone had no or little effect on HPAEC tubule formation (Figure 6A&B). To further support our finding of a role for endogenously released ET-1 in BMP-9 stimulated tubule formation, we investigated the effect of p38 MAPK inhibition using BIRB796 (1 µM) and Smad1 and 4 knock-down. In line with our previous results, p38 MAPK inhibition and Smad1 knock-down inhibited BMP-9 induced tubule formation, whilst knock-down of Smad4 had no effect (Figure 7).

### Role of endogenously released ET-1 in BMP-9-induced migration

**Figure 8:**
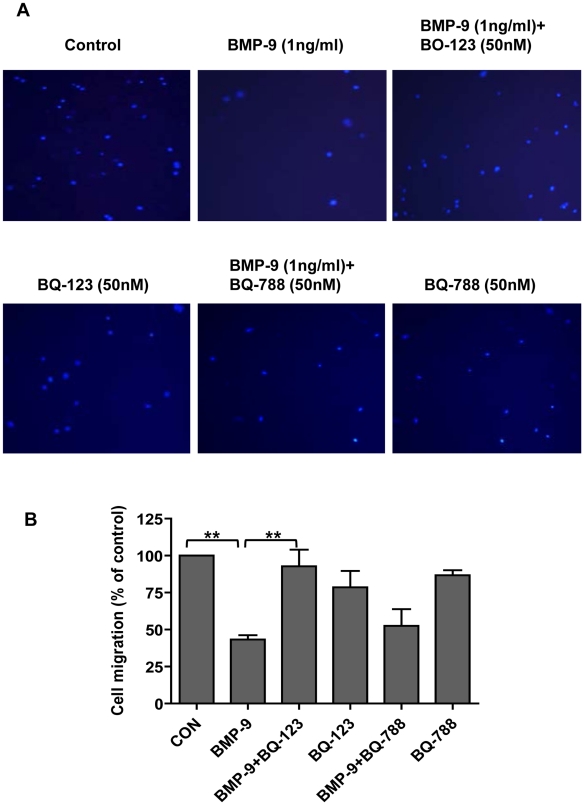
Effect of BMP-9 induced ET-1 on HPAEC migration. HPAECs were seeded onto transwell inserts and migration determined as described in the materials and methods section. Representitive images of 3 independent experiments of cells (nuclei identified with DAPI staining) migrated to the lower chamber are shown in (A). Migrated cells, under the conditions described shown, were counted and expressed graphically, as the percentage of the control in (B). Data are presented as mean ± SEM, n = 3. **p<0.01.

To further investigate a functional role of BMP-9 stimulated endogenous ET-1 release, we investigated the effect of BMP-9 on HPAEC migration using the Transwell migration assay. After 4 hrs BMP-9 (1 ng/ml) alone inhibited endothelial cell migration by 56.8±2.9% versus medium alone. This inhibition was almost entirely abolished by BQ-123, but not by BQ-788; neither inhibitor alone affecting migration (Figure 8A&B).

## Discussion

The main findings of this study are: (1) BMP-9 induced prepro ET-1 mRNA transcription and mature peptide release in HPAECs are dependent on Smad1 signalling but independent of co-Smad4; (2) p38 MAPK signalling is necessary for BMP-9 induced ET-1 release; (3) BMP-9 stimulated ET-1 release is only partially mediated through BMPR II and ALK1; (4) BMPR II knock-down increases basal prepro ET-1 mRNA transcription and mature peptide release and (5) BMP-9 induced endogenous ET-1 release is involved in BMP-9 stimulated HPAEC tubule formation and inhibition of HPAEC migration. Taken together these results support a role for BMP-9 induced ET-1 release in endothelial cell stability but not in the pathogenesis of PAH. However, BMPR II dysfunction appears important in regulating basal ET-1 levels and so may be important in the pathogenesis of PAH.

Although we and others have demonstrated BMP-9 induced ET-1 release from endothelial cells, this is the first study to determine the signalling pathways involved [21], [22]. Unsurprisingly, addition of BMP-9 to HPAECs resulted in phosphorylation of Smad1/5 and Smad2/3 pathways. However, after successful knock-down of the ‘common pathway’ Smad4 (confirmed by at least 80% knock-down of mRNA and protein) we found no effect on either prepro ET-1 mRNA or mature peptide release, whereas Smad1 knock-down consistently attenuated the induction of prepro ET-1 mRNA transcription by BMP-9. Furthermore, Smad 2 knock-down had no effect on prepro ET-1 mRNA transcription. Taken together, our results would suggest that BMP-9 stimulated ET-1 release does not use classical Smad signalling, but instead is dependent on Smad1 and independent of Smad4. Indeed, Smad4 independent signalling has been previously reported. For example, TGF-β mediated differentiation of haemopoietic/progenitor stem cells is dependent on binding of transcriptional intermediary factor 1γ (TIFI 1γ) to a phosphorylated Smad2/3 complex [27] whereas TGF-β mediated keratinocyte proliferation and differentiation involves Smad4 independent binding of IκB kinase (IKK)α to Smad2/3 [27], [28]. Furthermore, BMP-4 mediated sympathetic nervous system development in the mouse embryo is partly dependent on Smad4 independent, Smad 1/5/8 dependent activation of the paired-like homeobox transcription factor, Phox2β [29].

We found that both transcription of prepro ET-1 mRNA and release of mature peptide are dependent on p38 MAPK phosphorylation as determined by pharmacological inhibition using the compounds SB203580 and BIRB796, as well as upstream inhibition of the pathway using the TAK-1 inhibitor (5Z)-7-Oxozeanol. Indeed, MAPKs have long been implicated in TGF-β1 and BMP signalling; BMP-2, -4, -6 and -7 can signal through p38 MAPK dependent, Smad independent pathways [30], [31]. We found that HPAECs had a constitutive level of p38 MAPK phosphorylation with only a minor increase in phosphorylation with BMP-9 stimulation. We interpret these results as the need for a constitutive level of p38 MAPK phosphorylation for Smad1 induced prepro ET-1 mRNA transcription; BMP-9 induced increase in p38 MAPK phosphorylation *per se* is unlikely to be relevant. Of course, we realize a limitation in our pharmacological approach in that SB203580, BIRB796 and the TAK-1 inhibitor may be inhibiting alternative kinase pathways [32], [33] in addition to p38 MAPK, which are involved in co-operation with Smad1 driven signaling. However, BIRB796 has recently been reported to be highly specific for p38 MAPK, with an affinity greater than ten times than that for other kinases [26]. Crosstalk between MAPKs and Smad networks has been previously reported in various cell types. Overexpression of Smad6 or Smad7 can inhibit BMP-2 induced p38 MAPK phosphorylation, whilst receptor-Smads have MAPK consensus sites allowing differential phosphorylation by MAPKs [4]. In a lung cancer cell-line model, p38 MAPK activation was required for full activation of the transcriptional potential of Smad1/5, after treatment with BMP-4. Importantly, and of relevance to our study, SB203580 did not affect phosphorylation of Smad 1/5 (although not directly tested in our study). The mechanism involved p38 MAPK dependent co-operation of the transcription factor Sp1 with Smad1/5 on P16 and P21 promoters [5]. We would therefore hypothesize that a similar mechanism may be occurring in BMP-9 stimulated HPAECs. In further support of this mechanism, Rodriguez-Pascual *et al* have demonstrated that TGF-β stimulated prepro ET-1 mRNA transcription in bovine aortic endothelial cells requires co-operation of Smad2/3 and AP-1 on the prepro ET-1 promoter [34]. Finally, Morikawa *et al.* have reported for the first time Smad1/5 chromatin immune-precipitation (ChIP)-sequencing in human umbilical vein endothelial cells (HUVECs). It appears that Smad1/5 binding occurs mainly outside known target gene promoter regions. The authors identified, for the first time, a Smad1/5 binding motif in mammals (GC-SBE). They found that both GC-SBE and the canonical smad binding elements (SBE) affect binding affinity for the Smad complex [35]. The exact mechanisms involved in BMP-9 stimulated ET-1 release in HPAECs including Smad1 CHIP analysis are now the subject of further investigation.

We also demonstrated that BMP-9 induction of ET-1 release is only partially mediated by the type I receptor ALK1 and the type II receptor BMPR II. These findings are not surprising given previous studies suggesting redundancy of type I and type II receptors in endothelial cells. Indeed, Upton *et al* have recently demonstrated that in HPAECs, BMP-9 can signal through ALK1/BMPR II or ALK1/Actr II complexes, to activate Smad1/5 and Smad2 [2]. Our results would suggest that on knock-down of BMPR II, signalling may proceed via Actr II, although we did not directly look for this in the present study. In addition, and of potential importance, we found that knock-down of BMPR II caused a significant increase in basal ET-1 release and prepro ET-1 mRNA expression in HPAECs. This finding is potentially important as loss of BMPR II function appears widespread in PAH, where plasma and tissue ET-1 levels are also elevated [19], [36]; these findings suggest a mechanism by which ET-1 is elevated in PAH. In support of our findings is the recent observation by Talati *et al* that macrophages from patients with BMPR II mutations have an up-regulated ET-1 system, associated with reduction in ET_B_ receptor levels [37]. Clearly, increased ET-1 production by the vascular endothelial cells is likely to be more relevant to overall vascular remodeling, but concordance in these findings supports a clinically relevant interaction between BMPR II dysfunction and the ET-1 system and warrants further investigation. Furthermore, the partial dependence on BMPR II receptor function for BMP-9 induced, increased ET-1 release, would suggest that BMP-9 induced ET-1 release is not the mechanism for raised ET-1 production by endothelial cells in human PAH.

We found that BMP-9 enhanced tubule formation and inhibited cell migration in HPAECs, supporting the role for BMP-9 in enhancing vascular stability [23]. Both of these effects were dependent on endogenous ET-1 acting via the ET_A_ receptor. Further support for endogenous ET-1 mediating these effects was demonstrated by dependence on p38 MAPK and Smad1 pathways (but not Smad 4) for BMP-9 stimulated tubule formation, in line with our previous signalling results. Although BMP-9 induced tubule formation is a novel observation, BMP-9 does promote vessel formation from allantoic explants at low concentrations (4 ng/ml) [38], whilst inhibiting endothelial sprouting from embryoid bodies at higher concentrations (10 and 100 ng/ml), suggesting a concentration dependent effect [23]. Equally, BMP-9 has been shown to both inhibit and induce cell proliferation [39], [40], which may again depend on the concentration used, as well as cell type differences [2], [39]. Indeed, in our system using lower (and more physiological) BMP-9 concentrations, we found no effect on HPAEC proliferation. Finally, other BMPs such as BMP-2 and BMP-4 can also stimulate tubule formation [41]. Our finding of BMP-9 induced inhibition of endothelial cell migration confirms the findings of Lee *et al*. In their study BMP-9 inhibition of migration was ALK1 dependent and enhanced by CK2β, the regulatory subunit of protein kinase CK2 [42]. Overall, our data and that of others, suggest that BMP-9 is important in processes pertinent to new vessel formation and stabilisation. Indeed, disruption in ALK1 mediated signalling in endothelial cells is known to cause disordered angiogenesis, as seen in hereditary haemorrhagic telangiectasia (HHT) where mutation in ALK1 has been described [43], [44]. Disruption of BMP-9 signalling through ALK1 may therefore be important in the development of HHT related vascular changes.

Whereas ET-1 has been shown to promote endothelial tubule formation [45], [46], in agreement with our findings, it is also generally known as a chemo-attractant, promoting migration [46]–[48]. Furthermore, migration of HUVECs, stimulated by ascitic fluid from patients with ovarian cancer (known to contain high levels of ET-1 and vascular endothelial growth factor (vEGF), was more effectively blocked by the ET_B_ receptor antagonist, BQ-788 (55%) than the ET_A_ receptor antagonist, BQ-123 (25%) [16]. In our cells, the opposite was found, in that migration was ET_A_ receptor dependent. However, in support of our findings, Rodriguez-Pascual *et al.* reported in bovine aortic endothelial cells that TGF-β had anti-migratory actions, due to endogenous ET-1 production, being reversed by the mixed endothelin receptor antagonist bosentan [49]. Furthermore, in that study, the anti-migratory effect of ET-1 was dependent on active TGF-β signaling, suggesting that ET-1 was acting in conjunction with other TGF-β stimulated mediators. It is possible that a similar situation exists for BMP-9 in our system. Finally, although it is generally believed that the ET_A_ receptor is mainly active on vascular smooth muscle and not endothelial cells, there are several recent papers reporting ET_A_ expression and activity in endothelial cells of various origins, including pulmonary vascular endothelial cells in patients with idiopathic PAH [50]–[54]. Indeed, we confirmed the expression of ET_A_ and ET_B_ receptor mRNA in HPAECs, although no change in mRNA expression was found with BMP-9 stimulation (data not shown).

In conclusion, our results demonstrate that BMP-9 induced ET-1 release in HPAECs is partially mediated through the ALK1/BMPR II receptor complex, in a Smad1 and p38 MAPK dependent manner. BMP-9 also promotes tubule formation, and inhibits HPAEC migration, both effects blocked by ET_A_ receptor antagonism, suggesting an important functional role for BMP-9 stimulated endogenous ET-1. These findings support the concept of BMP-9 being a circulating factor affecting angiogenesis and endothelial stabilisation. However, the dependence of this effect on BMPR II signalling argues against an important role for BMP-9 as a source of increased endothelial ET-1 production in human PAH. In fact, such signalling may be more relevant for diseases such as HHT. Importantly, BMPR II dysregulation *per se* may increase basal endothelial cell ET-1 release, suggesting a potential role in the pathogenesis of PAH, and warranting further investigation.

## Materials and Methods

### Cell culture

Primary human pulmonary artery endothelial cells (HPAECs: Lonza, Wokingham, UK) maintained in complete endothelial cell growth medium-2 (EGM-2) were used at passages 3–8.

### Kinase pathways inhibition

After quiescence for 16 hrs in EGM-2 without hydrocortisone and 0.1% FBS, HPAECs were pre-treated with the mitogen activated protein kinase (MEK)1/2/ERK inhibitor UO126 (1, 3 and 10 µM), the JNK inhibitor, SP600125 (1, 3 and 10 µM), the p38MAPK inhibitors, SB203580 (1, 3 and 10 µM) (Cell Signaling, Beverley, MA, USA) and BIRB796 (1 µM), or the transforming growth factor-β activating kinase (TAK-1) inhibitor (5Z)-7-Oxozeanol (0.5 µM. Calbiochem, San Diego, CA, USA), or vehicle controls for 1 hr. BMP-9 (R&D Systems, Abingdon, UK) was added to give a final concentration of 1 ng/ml for a further 24 hrs.

### Endothelin ELISA

After quiescence for 16 hrs in EGM-2 without hydrocortisone and 0.1% FBS, HPAECs were treated with either BMP-2, -4, -6 or -9 for 24 hrs. After treatment, media were collected and cleared by centrifugation at 10,000×g for 1 min. ET-1 concentration in supernatants was measured by enzyme-linked immunosorbent assay (ELISA) according to the manufacturer's instruction (R&D Systems, Abingdon, UK) as previously described [55].

### Cell Proliferation

After aspiration of supernatants, cell viability was determined by MTT assay or cells were frozen at −80°C and analyzed by the CyQUANT assay (Sigma-Aldrich, Poole, UK), as per the manufacturer's protocol.

### Small interfering RNA silencing

Small interfering RNA knock-down was carried out as described previously [2]. In brief, HPAECs were transfected with 20 nM of siRNA duplexes for Smad4 or 10 nM for ALK1, Smad1, Smad2, Smad5 (OnTARGET-Plus siGenome, Dharmacon™, Lafayette, CO, USA) or BMPR II (Dharmacon Smartpool®), or non-targeting control pool siRNA in complex with DharmaFECT1™ transfection reagent (DH1) (4 µl/well for 6-well plate or 8.75 µl/dish for 6-cm dish) in OptiMem I (Invitrogen, Paisley, UK) for 4 hrs.

### RNA extraction and real time qRT-PCR

Total RNA (1 µg) was transcribed to cDNA using L-AMV reverse transcriptase (Invitrogen, Paisley, UK) or a high capacity cDNA reverse transcription kit (Applied Biosystems, Paisley, UK) according to the manufacturer's instructions. Real-time quantitative PCR was performed using SYBR green master mix (Qiagen, West Sussex, UK) or SYBR® Green Jumpstart™ Taq Readymix™ (Sigma, Poole, UK) on a Corbett Rotor-Gene 6000 (Qiagen, West Sussex, UK) or an ABI StepOne Plus™ (Applied Biosystems, Paisley, UK). The relative expression of target genes was quantified using the ΔΔCt method normalized to the average expression of two housekeeping genes (β-actin and GUSB) [56]. The sequences for ET-1, ALK1, BMPR II, Smad1, Smad5, Smad4, Id-1, β-actin and GUSB primers are described previously [2], [57].

### Immunoblotting

20 µg of total protein (30 µg for p38 MAPK and Smad2) from whole cell lysates was separated on SDS-PAGE and transferred to nitrocellulose membranes or polyvinylidene fluoride membranes (GE Healthcare, Buckinghamshire, UK). Membranes were first probed with anti-phospho-p38, phospho-ERK1/2, phospho-JNK, phospho-Smad1/5, phospho-Smad2, Smad1, Smad4, Smad5 (Cell Signaling, Beverley, MA, USA), BMPR II (BD Bioscience, Oxford, UK) or ALK1 (kindly provided by Professor D.A. Marchuk, Duke University Medical Center) then stripped with 50 mM Tris, pH 8.0, 150 mM NaCl, 2%SDS and 100 mM β-mecaptoethanol for 1 hr at 55°C, and re-probed with total p38 MAPK, ERK1/2, JNK, Smad1/5 and Smad2 (Cell Signaling, Beverley, MA, USA), α-tubulin or β-actin (Sigma, Poole, UK) to confirm equal protein loading). Membranes were developed using ECL (GE Healthcare, Buckinghamshire, UK). The intensity of the bands was quantified using Image J (NIH).

### Tubule formation assay

HPAECs (5×10^4^/well) or HPAECs transfected with siRNA (CP, Smad1 or Smad4, 68 hrs post-transfection) were seeded on 8-well slides coated with growth factor reduced matrigel (200 µl/well: BD Biosciences, Oxford, UK) and incubated for 4 hrs in the presence or absence of BMP-9 (1 ng/ml), and various concentrations of the ET_A_ inhibitor BQ-123 or the ET_B_ inhibitor, BQ-788 (10 nM–2 µM, Tocris Bioscience, Bristol, UK), or 1 µM BIRB796.

### Cell migration assay

Serum starved HPAECs (5×10^4^ cells) were added to the upper chamber of a 24-well insert (8 µm pore, Transwell®) pre-coated with gelatin (20 µg/insert) in the presence of BQ-123, BQ-788 or vehicle control. Starvation medium (600 µl) with or without BMP-9 (1 ng/ml), BQ-123, BQ-788 or vehicle control was added to the lower chamber. Plates were incubated at 37°C for 4 hrs. After incubation, cells in the upper chambers were removed with cotton buds. Cells on the lower chamber membranes were fixed with 3.7% formaldehyde for 5 min, the membrane was then cut and mounted between a slide and a coverslip in ProLong antifade mounting solution containing DPI (Invitrogen, Paisley, UK). Images were taken using a Zeiss Fluorescence microscope.

### Statistical analysis

Experimental data are presented as mean ± SEM. BMP dose response experiments were analyzed using Friedman's non-parametric repeated measures test with Dunn's post test correction. The remaining data were analyzed using Kruskal-Wallis non-parametric analysis with Dunn's Multiple Comparison Test or two-way ANOVA with Bonferroni Post test, using GraphPad Prism4.

## Supporting Information

Figure S1 **ET-1 release from HLMVECs in response to BMP-9 stimulation.** HLMVECs were grown to confluence on 96-well plates and starved for 16 hrs. Cells were then stimulated with increasing concentrations of BMP-9. Supernatants were collected at 24 hrs of treatment and ET-1 level assayed by ELISA. Data are presented as mean ± SEM. n = 6 * p<0.05.
(TIF)

Figure S2 **Re-expression of Smad1 and BMPR II in HPAECs rescues the effect of Smad1 and BMPR II siRNA silencing respectively.** HPAECs were grown to confluence on 96-well plates and transfected with siRNAs for 4 hrs and left over-night. Cells were then infected with viral particles encoding mouse Smad1 (A), BMPR II (B) or GFP for 16 hrs. After viral transduction, cells were starved for a further 16 hrs and then stimulated with 1 ng/ml of BMP-9 for 24 hrs. Supernatants were then collected and ET-1 level assayed by ELISA. Data are presented as mean ± SEM, n = 3. *** p<0.001, # p>0.05.
(TIF)

Figure S3 **Effects of CP, DH1, Smad1 and BMPR II siRNA transfection on the morphology of HPAECs.** HPAECs were seeded onto coverslips and transfected with siRNAs as described in the materials and methods section. Representitive cell morphology is shown. Red: F-actin cytoskeleton identified with TRITC-phalloidin staining; Blue: nuclei identified with DAPI staining.
(TIF)

Text S1 **Materials and methods.**
(DOC)
