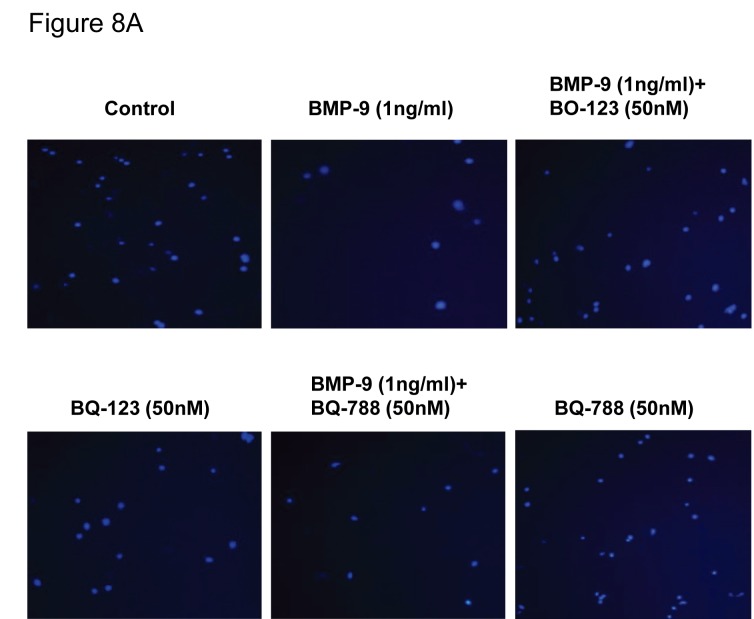# Correction: BMP-9 Induced Endothelial Cell Tubule Formation and Inhibition of Migration Involves Smad1 Driven Endothelin-1 Production

**DOI:** 10.1371/annotation/71fb82bd-fd34-497e-a887-b1167f43570a

**Published:** 2013-02-28

**Authors:** John E. S. Park, Dongmin Shao, Paul D. Upton, Patricia deSouza, Ian M. Adcock, Rachel J. Davies, Nicholas W. Morrell, Mark J. D. Griffiths, Stephen J. Wort

The authors have identified errors in relation to two of the figures in the article which they would like to address via this Correction. The errors identified do not affect the results of the experiments or the conclusions reported in the article.

Figure 1B. On reviewing the data for the BMP-4 experiment we identified a transcription error whereby data from one experiment was included twice but in the wrong order. The incorrect line of data has been removed and replaced with the original, intended data. The data has been re-analyzed and a new figure constructed (see corrected Figure 1B). There is no difference in the overall results, the overall n number remains unchanged (n=7).

Figure 8A. The image representative for the condition BMP-9 and BQ788 was duplicated by accident and included as the BQ788 alone image. We provide a true representative image for BQ788 alone in a new figure (see corrected Figure 8A). 

Fig 1B: 

**Figure:**
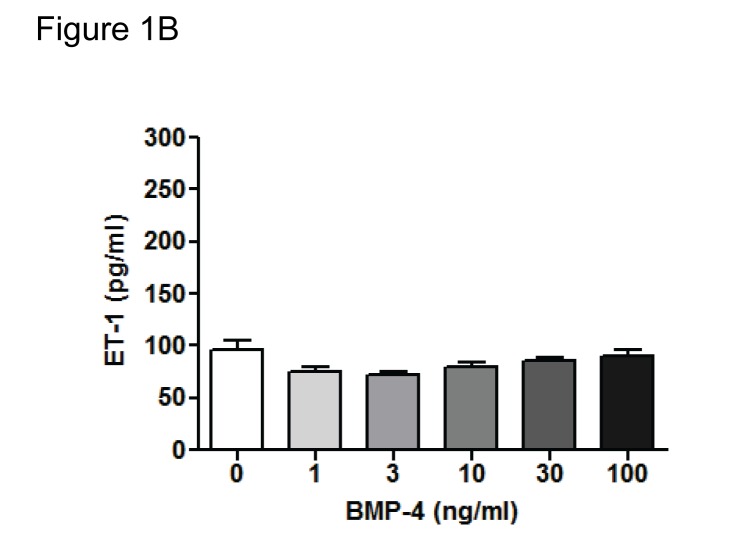


Fig 8A: 

**Figure:**