# Supplementary figures and images for: BMP-9 Induced Endothelial Cell Tubule Formation and Inhibition of Migration Involves Smad1 Driven Endothelin-1 Production
Source: PLoS One. 2012 Jan 27;7(1):e30075. doi: 10.1371/journal.pone.0030075 (PMC3267722; doi:10.1371/journal.pone.0030075)

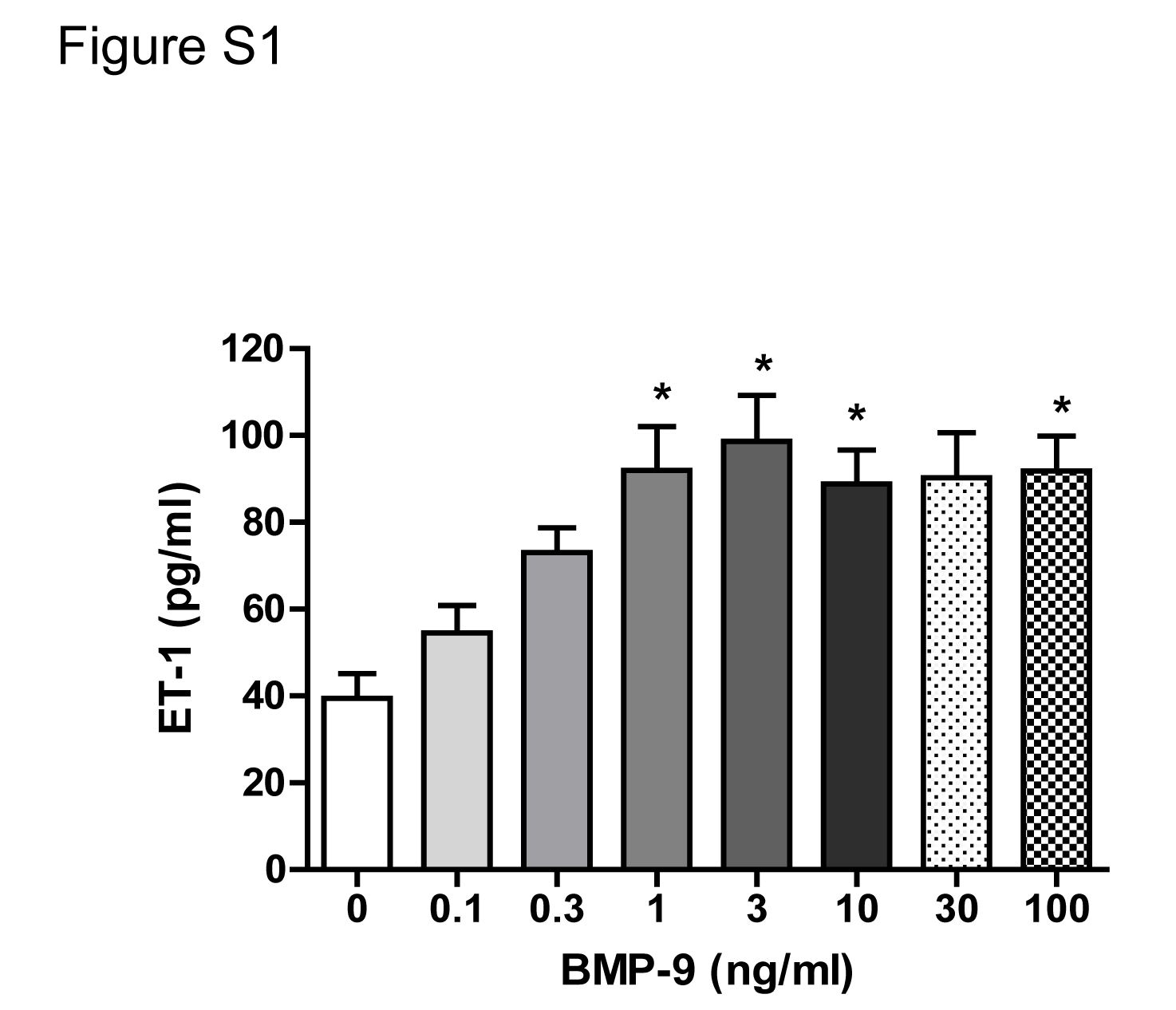

Supplement: Figure S1 — ET-1 release from HLMVECs in response to BMP-9 stimulation. HLMVECs were grown to confluence on 96-well plates and starved for 16 hrs. Cells were then stimulated with increasing concentrations of BMP-9. Supernatants were collected at 24 hrs of treatment and ET-1 level assayed by ELISA. Data are presented as mean ± SEM. n = 6 * p<0.05. (TIF) [file pone.0030075.s001.tif]

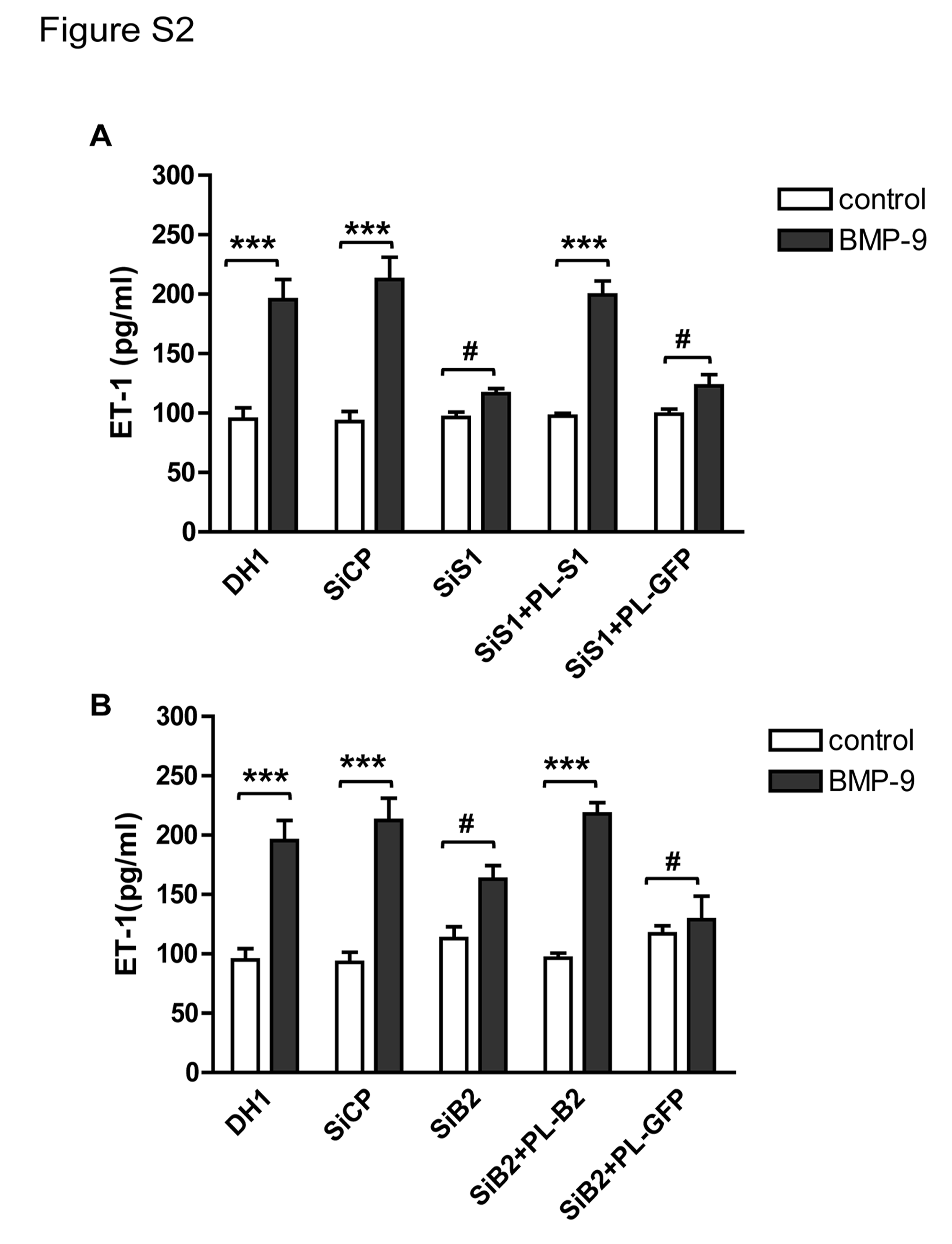

Supplement: Figure S2 — Re-expression of Smad1 and BMPR II in HPAECs rescues the effect of Smad1 and BMPR II siRNA silencing respectively. HPAECs were grown to confluence on 96-well plates and transfected with siRNAs for 4 hrs and left over-night. Cells were then infected with viral particles encoding mouse Smad1 (A), BMPR II (B) or GFP for 16 hrs. After viral transduction, cells were starved for a further 16 hrs and then stimulated with 1 ng/ml of BMP-9 for 24 hrs. Supernatants were then collected and ET-1 level assayed by ELISA. Data are presented as mean ± SEM, n = 3. *** p<0.001, # p>0.05. (TIF) [file pone.0030075.s002.tif]

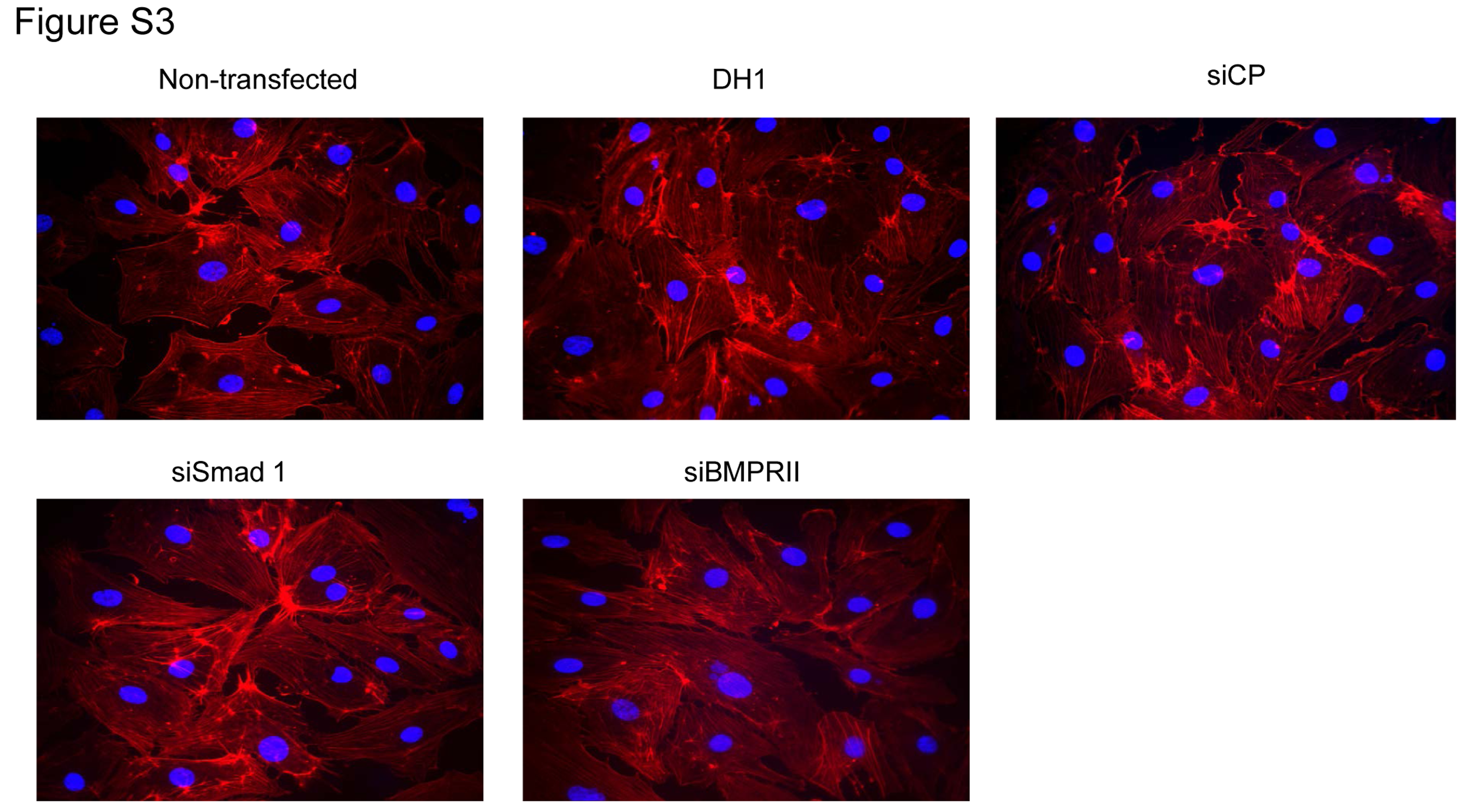

Supplement: Figure S3 — Effects of CP, DH1, Smad1 and BMPR II siRNA transfection on the morphology of HPAECs. HPAECs were seeded onto coverslips and transfected with siRNAs as described in the materials and methods section. Representitive cell morphology is shown. Red: F-actin cytoskeleton identified with TRITC-phalloidin staining; Blue: nuclei identified with DAPI staining. (TIF) [file pone.0030075.s003.tif]
