# Supplementary material for: BMP-9 Induced Endothelial Cell Tubule Formation and Inhibition of Migration Involves Smad1 Driven Endothelin-1 Production
Source: PLoS One. 2012 Jan 27;7(1):e30075. doi: 10.1371/journal.pone.0030075 (PMC3267722; doi:10.1371/journal.pone.0030075)
Supplement: Text S1 — Materials and methods. (DOC) [file pone.0030075.s004.doc]

**Text S1**

**Materials and methods:**

**Cell culture**

Primary human lung microvascular endothelial cells (HLMVECs: Lonza, Wokingham, UK) were maintained in complete endothelial cell growth medium MV2 (EGM MV2). Experiments were performed with cells between passages 3-6.

**Endothelin ELISA**

After quiescence for 16 hrs in EGM MV2 without hydrocortisone and 0.1% FBS, HLMVECs were treated with increasing concentrations of BMP-9 (0-100ng/ml) for 24 hrs. After treatment, media were collected and cleared by centrifugation at 10,000xg for 1 min. ET-1 concentration in supernatants was measured by Enzyme-linked immunosorbent assay (ELISA) according to the manufacturer’s instruction (R&D Systems, Abingdon, UK) as previously described [55].

**Immunofluorescence microscopy**

HPAECs (3x10^4^ cells) were seeded onto 13-mm coverslips and were incubated at 37ºC for 48 hrs. Cells were transfected with negative control siRNA (CP), DH1, Smad1 and BMPR II siRNA. 72 hrs after transfection, cells were fixed with 3.7% formaldehyde for 5 min, and extracted with 0.2% Triton in PBS for 10 min. After blocking with 1% BSA in PBS, cells were stained with 1µg/ml of TRITC-phalloidin (Sigma Poole, UK) for 45 min, and washed 5 times with PBS for 5 min each. After washing, the coverslips were mounted onto slides in ProLong antifade mounting solution containing DPI (Invitrogen, Paisley, UK). Images were taken using a Zeiss fluorescence microscope.

**Lentiviral constructs and virus transduction**

Lentiviral constructs encoding mouse Smad1 (EX-Mm03732-Lv105) or BMPR II (EX-Mm01452-Lv105) were purchased from GeneCopoeia (Rockville, USA). Retroviral particles were produced using a Lenti-Pac HIV Expression Packaging Kit according to the manufacturer’s recommendations with the following modifications: culture flasks (175 ml) were plated with 4 x 10^6^ 293T/17 cells (kind gift from Dr. Uta Griesenbach, NHLI, Imperial College) and maintained in complete DMEM (DMEM containing 10% FBS/penicillin/streptomycin) for 48 hrs prior to transfection. Each flask was transfected with a total of 7.5 μg of vector DNA (5 μg of Viral Power Mix and 2.5 μg of lentiviral vector) using Endofectin-lenti (GeneCopoeia). The next day, producer cells were fed with DMEM containing 5% FBS and Titeboost (1:500) (conditional medium). After 48 hrs, conditioned media (CM) containing viral particles were collected and spun at 2,000 rpm for 10 min, and kept at -80ºC in aliquots. HPAECs were plated at 7.5x10^3^ cells/well in 96-well plates and siRNA silencing performed with either Smad1 or BMPR II for 4 hrs as previously described. Cells were rested overnight. Then 100 μl of CM was added to the wells, followed by the addition of polybrene to a final concentration of 5μg/ml. The plate was spun at room temperature at 1,000 rpm for 60 min and was then returned to the incubator overnight. After 16 hrs starvation, HPAECs were then stimulated with 1 ng/ml BMP-9 for 24 hrs. Finally, supernatants were collected and ET-1 level was analysed by ELISA as described previously.
